# Immunosuppressive effect and global dysregulation of blood transcriptome in response to psychosocial stress in vervet monkeys (*Chlorocebus sabaeus*)

**DOI:** 10.1038/s41598-020-59934-z

**Published:** 2020-02-26

**Authors:** Anna J. Jasinska, Ivona Pandrea, Tianyu He, Cassandra Benjamin, Maurice Newton, Jen Chieh Lee, Nelson B. Freimer, Giovanni Coppola, James D. Jentsch

**Affiliations:** 1 Center for Neurobehavioral Genetics, Semel Institute for Neuroscience and Human Behavior, The University of California Los Angeles, California, USA; 2 Institute of Bioorganic Chemistry, Polish Academy of Sciences, Poznan, Poland; 3 Department of Pathology, School of Medicine, University of Pittsburgh, Pittsburgh, Pennsylvania USA; 4 St. Kitts Biomedical Research Foundation, St. Kitts, West Indies Saint Kitts and Nevis; 5 Department of Neurology, The University of California Los Angeles, California, USA; 6 Department of Psychology, Binghamton University, Binghamton, NY 13902 USA

**Keywords:** Behavioural genetics, Stress and resilience

## Abstract

Psychosocial stressors - life events that challenge social support and relationships - represent powerful risk factors for human disease; included amongst these events are relocation, isolation and displacement. To evaluate the impact of a controlled psychosocial stressor on physiology and underlying molecular pathways, we longitudinally studied the influence of a 28-day period of quarantine on biomarkers of immune signalling, microbial translocation, glycaemic health and blood transcriptome in the wild-born vervet monkey. This event caused a coordinated, mostly transient, reduction of circulating levels of nine immune signalling molecules. These were paralleled by a massive dysregulation of blood transcriptome, including genes implicated in chronic pathologies and immune functions. Immune and inflammatory functions were enriched among the genes downregulated in response to stress. An upregulation of genes involved in blood coagulation, platelet activation was characteristic of the rapid response to stress induction. Stress also decreased neutrophils and increased CD4 + T cell proportions in blood. This model of psychosocial stress, characterised by an immune dysregulation at the transcriptomic, molecular and cellular levels, creates opportunities to uncover the underlying mechanisms of stress-related diseases with an immune component, including cardiovascular diseases and susceptibility to infections.

## Introduction

Psychosocial stress is a key risk factor for numerous chronic diseases, particularly cardiovascular (e.g., hypertension and atherosclerosis), metabolic (e.g., diabetes), and neuro-psychiatric morbidity (e.g., depression and anxiety, substance use disorders, neurodegenerative disease and psychosis)^1–8^. Stress responses constitute an adaptive strategy for maintaining bodily homeostasis in physically or psychologically challenging situations and are, in part, orchestrated by the hypothalamus-pituitary-adrenal (HPA) axis and controlled by its end products, glucocorticoids, through a negative feedback loop. However, prolonged exposure to stress, chronic activation of the HPA, and continuous secretion of glucocorticoids exert maladaptive effects and lead to the wide range of health complications mentioned above. Cortisol is regarded as a major mediator of a range of stress responses affecting many body systems, including the suppression of HPA activity, regulation of immune functions, glucose metabolism, and gene expression across different organ systems and, if chronically elevated, is a key driver of stress-related pathologies.

Cortisol is commonly used physiological indicator of an individual’s response to stress;^9,10^ notably, however, this relationship is moderated by multiple biological and environmental variables (including - but not limited to - age, circadian rhythm, nutritional status and other exposures) and the tissue or other biological resource used for measurements^11–15^. Additionally, the magnitude of the cortisol response to stress can habituate or sensitize with repeated or prolonged exposure to the same or different stressors, effects mediated through multiple mechanisms, including altered negative feedback at the level of brain and pituitary, changes in the activity of pro-stress circuitry in brain, and complex learning mechanisms^16,17^. Consequently, the interpretation of cortisol levels as biomarker of chronic stress should therefore be treated with caution.

Importantly, not all individuals respond equivalently to the same stress challenge, nor do they show the same stress-induced pathology. Inter-individual variability in stress responsivity and susceptibility to stress-triggered diseases is shaped by multiple factors, including the variety of stressors encountered in everyday life, other environmental factors, and comorbid disorders. These confounding variables are difficult to control or even account for (such as diet, medications, lifestyle, etc.) in human cohorts. Many of these factors could be better controlled in an experimental setting using animal models, among which Non-Human Primates (NHPs) stand out as most closely reproducing the biology of the cognitive, metabolic, and immune processes observed in humans because of their close phylogenetic relatedness^18^.

In naturally living NHPs, a major form of psychosocial stress derives from the major rearrangements in social support and hierarchy that often occur across the lifespan^19^. In a laboratory setting, some of these stressful events can be mimicked when animal relocation between facilities and/or social group rearrangements occur, creating a model system for studying responses to a defined psychosocial stress. Relocations were previously associated with increased cortisol levels in Garnett’s bushbaby^20^ and with behavioral consequences such as self-injuries and sleep disturbances in rhesus macaques^21^, demonstrating that they can trigger both neuroendocrine and behavioral stress responses in NHPs serving as a model for stress studies. Relocation has also been observed to affect hematological, clinical chemistry, and immunological parameters in chimpanzees^22^. Psychosocial stressors in NHPs, such as experimentally enforced low position in a social hierarchy/dominance rank, have lasting yet plastic (i.e., reversible) effects on gene expression in blood and also affect blood cell composition, diminish negative feedback on the HPA, and modulate chromatin accessibility^23,24^. Overall, investigations involving removal from a current group, experimental manipulation of social rank, and early life adversities (e.g., adverse prenatal environment and early postnatal rearing environments) demonstrated both that the NHPs share many characteristics of the stress response with humans^25^ and that social environment has an effect on health and gene expression in both humans and NHPs^26,27^.

To develop an NHP model of psychosocial stress (PS) exposure that would facilitate longitudinal studies of the effects of a uniform (standardized) stressor under controlled conditions, we turned to the vervet monkey (*Chlorocebus sabaeus*), an established NHP biomedical research model^28–30^. The vervet represented an ideal animal model for our study as it was previously reported to show significant neuroendocrine and behavioral responses to relocation between research centers^31,32^ and neuroanatomical alterations (in the hippocampus) upon relocation from the wild to a research facility^33^.

A small number of vervets from West Africa were introduced to the Caribbean Islands during the colonial era. The feral vervet population vastly expanded in the ecosystem due to the absence of natural predators. Wild-born vervet monkeys from the highly abundant population on St. Kitts Island are currently a source of animals for biomedical research in multiple local primate facilities^28^. After capture, wild-born monkeys are placed in a 28-day quarantine in the primate facility, during which they are sometimes housed individually and assessed for infectious agents and behavioral problems before being integrated to the colony. We hypothesized that the temporary disruption of social support following removal from the family troop and placement in solitary housing would be a meaningful psychosocial stressor that would exceed other stressors experienced thus far in juvenile individuals and, therefore, that this setting would represent a natural model for stress studies.

Here, we present the neuroendocrine, immune, metabolic, and transcriptomic responses of 15 Kittitian juvenile vervet males subjected to conditions closely mimicking the standardized quarantine procedures. We demonstrate that the quarantine-related stress significantly dysregulated the immune system, blood transcriptome, and blood cell composition, supporting our hypothesis that the quarantine setting represents a natural model system for studies on PS in the vervet monkey.

## Results

### Biochemical responses to PS

During the period of relocation and quarantine, hair cortisol increased from 68.62 ± 83.03 pg/mg (before quarantine at day 0 (Tp1)) to 96.49 ± 62.65 pg/mg (after quarantine on day 28 (Tp6)) (Supplementary Figure 1). A Wilcoxon Signed-Rank Test indicated that hair cortisol level was statistically significantly increased after the quarantine Z = 1.69, p < 0.0455 (one tail), when the entire group of 15 individuals was tested. We observed large inter-individual variability in baseline cortisol levels because of two individuals, VSC00010 and VSC00014, that exhibited high baseline cortisol levels. These individuals came from different regions of St. Kitts Island and did not show any visible signs of old or fresh injuries, poor nutrition, or other health problems that could explain the elevated cortisol levels or additional sources of stress. It is possible that these individuals experienced unknown social circumstances in their troops^34^ that contributed to their high cortisol levels. After quarantine, there were two upper outliers that stood out due to very high hair cortisol levels: VSC00010 and VSC00015 (which was the only individual subjected to quarantine during Hurricane Danny, a possible additional stressor). After exclusion of these three individuals with outlying individuals measurements (defined according to the 1.5 x IQR rule), we observed a significant increase in hair cortisol level from 39 ± 15.96 pg/mg on day 0 (Tp1) to 72.10 ± 30.69 pg/mg on day 28 (Tp6) after the quarantine (Wilcoxon Signed-Ranks test, Z = 2.57, p < 0.0051 [one tail]). This increase in cortisol during quarantine is consistent with the sustained activation of the HPA axis, implying that quarantine was a meaningful stressor and thereby demonstrating the validity of our PS model.

**Figure 1:**
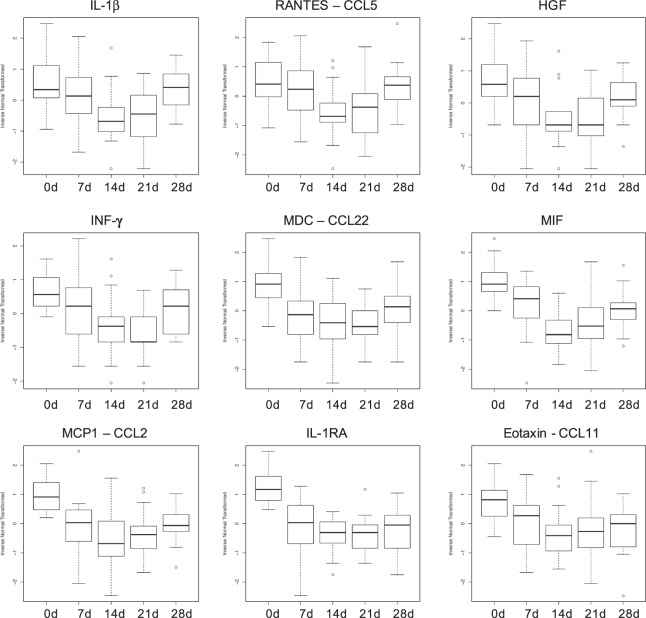
Circulating cytokine, chemokine, and growth factor levels (IL-1β, RANTES, Eotaxin, MCP1, HGF, IFN-γ, MDC, MIF, IL-1RA) at five experimental time points: day 0 (Tp1 i.e. baseline), day 7 (Tp3), day 14 (Tp4), day 21 (Tp5), and day 28 (Tp6).

Stress can induce a wide spectrum of alterations in immune indices associated with immunoprotective, immunopathological, and immunoregulatory/inhibitory effects^35^. Longitudinal assessment of circulating levels of 29 immune biomarkers revealed that mean levels of nine molecules (IL-1β, IL-1RA, IFN-γ, eotaxin, MCP1, MDC, MIF, RANTES, and HGF) showed changes across the experimental time points that were nominally significant (p < 0.05, Table 1). Immune biomarker molecules with typically pro-inflammatory effects (e.g., IL-1β and IFN-γ) as well such molecules with anti-inflammatory effects (e.g., IL1-RA and HGF) showed a coordinated decrease in response to the relocation and quarantine stressor (Fig. 1). The gradual decrease in average cytokine levels, which reached their maximum on day 14, is consistent with PS-induced suppression of the immune system. After day 14, average levels of RANTES and IL-1β evidenced recovery, suggesting progressive habituation to the stressor, while seven other biomarkers (Eotaxin, MCP-1, HGF, IFN-γ, MDC, MIF, IL-1RA) remained significantly different (P < 0.05) from the baseline at the end of quarantine period. Stress can induce immunoprotective (e.g., promote wound healing and eliminate infections), immunopathological, and immunoregulatory effects depending on the context and the duration of the stressor^36–40^. Typically, short-term stress lasting for minutes, hours, or days triggers adaptive mechanisms, preparing the body for challenges that may be imposed by stressor, such as wounds, injuries, or infections, through enhancing immune responses. However, long-term or repeated, intermittent stress lasting for weeks or months can suppress or dysregulate immune responses^38^. Based on the pattern in immune marker response, we can conclude that the applied quarantine conditions represented a long-term, persistent stressor that evoked immune suppression.

**Table 1:** Variability of immune, microbial translocation, and glycemic biomarkers.

Variable	ANOVA p-value	Tp6 vs. Tp1 p-value*	p-value for inclusion of random effect
sCD14	4.14E-01	6.00e-01	1.06E-01
LPS	4.19E-01	8.05e-02	2.26E-01
IL-1β	**8.49E-05**	5.36e-01	**1.17E-04**
IL-12	2.41E-01	9.18e-01	**1.33E-07**
RANTES	**1.67E-04**	6.41e-01	**5.11E-04**
Eotaxin	**2.07E-03**	**1.18e-03**	**3.22E-03**
MCP-1	**8.24E-06**	**5.41e-04**	**9.76E-03**
HGF	**3.75E-05**	**4.02e-02**	**8.74E-06**
IFN-γ	**3.48E-05**	**1.65e-02**	**3.56E-04**
MDC	**4.46E-04**	**3.14e-02**	**2.74E-02**
MIF	**1.73E-09**	**3.53e-04**	**2.40E-05**
IL-1RA	**1.50E-10**	**2.25e-06**	**4.33E-05**
IP-10	5.36E-01	6.20e-01	**2.18E-03**
IL-4	3.30E-01	1.870e-01	4.83E-01
A1c	1.26E-01	3.11e-01	**1.97E-02**
GHb	1.44E-01	2.09e-01	**2.76E-02**

**bold** face p-value < 0.05.

*paired t-test p-value.

Psychosocial stress can also increase the permeability of the intestinal barrier, causing the translocation of bacteria and antigens from the gut to the bloodstream and, in turn, activating pro-inflammatory responses^41–43^. To assess the potential effects of PS on the permeability of the gut epithelium and systemic inflammatory state, we investigated plasma levels of the microbial translocation biomarker sCD14, an indicator of monocyte activation, as well as levels of the bacterial endotoxin lipopolysaccharide (LPS). We did not observe significant changes in circulating levels of sCD14 or LPS (Supplementary Figure 2), suggesting that the animals maintained an intact mucosal barrier that prevented microbial translocation.

Given that emotional stress is a risk factor for the development of type 2 diabetes (T2D) in healthy individuals^44^ and aggravates outcomes in people already affected by T2D^45^, we predicted that PS would increase circulating levels of the glycemic biomarkers, A1C and GHb. We observed that neither of these glycemic biomarkers showed significant variability over the course of the PS (Supplementary Figure 3). The lack of effect on these glycemic measures may be related to the young age of our study subjects, considering that the risk of type II diabetes generally increases with older age^46,47^. Also, the 28-day exposure period may be too short to dysregulate the metabolic system and reflect only a fraction of the potential long-term alterations to hemoglobin glycation levels.

Individuals differ with respect to perception of and reaction to stressors, resulting in different susceptibilities to stress-related diseases. These differences are generally shaped by two factors: genetic background and environmental exposure. We anticipated that the well-controlled quarantine environment, where external influences were minimized, would make the inter-individual variability in stress responses due to genetic differences more apparent. We observed that, of nine stress-reactive immune markers, all - except IL-4 - showed significant inter-individual variability in the stress response (Table 1) and may represent candidate biomarkers with potential genetic contribution to stress reactivity for further studies.

### Massive dysregulation of blood transcriptome in response to the relocation stressor

Glucocorticoids have widespread effects on transcriptional regulation^48^. To assess stress-related changes in gene expression, we analyzed the RNA-seq transcriptomic profiles of the monkeys at three time points during the PS experiment: day 0 (Tp1, baseline conditions), day 3 (Tp2), and day 14 (Tp4). The latter two time points hypothetically represented short-term and long-term PS exposures, respectively. We employed two approaches to identify genes whose expression changed over time in response to the stressor: differential gene expression analysis, comparing expression between different experimental time points, and network analysis, identifying modules of co-expressed genes associated with experimental time points.

We observed significant changes in the expression of 3,393 genes on day 3 vs. day 0, 7,551 genes on day 14 vs. day 0, and 4,286 genes on day 14 vs. day 3 (FDR threshold of 0.05), corresponding with a total of 9,055 dysregulated genes (Supplementary Table 1). Considering an absolute log2 fold change of “1” or greater as the threshold, we observed significant dysregulation in the expression of 556 genes (205 genes up-regulated and 351 genes down-regulated) on day 3 vs. day 0, 2,304 genes (1,441 up and 863 down) on day 14 vs. day 0, and 863 genes (700 up and 163 down) on day 14 vs. day 3.

**Figure 2:**
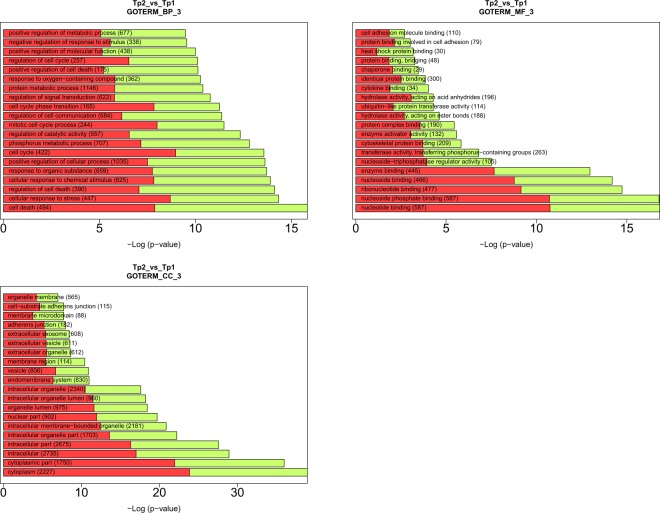
Gene Ontology (GO) enrichment analysis among stress-related genes genes based on the David tool. The red and green bars correspond to the proportion of up- and down-regulated genes.

Many of the genes that showed a strong dysregulation in response to the PS (log2FC below −3 or above 3) are involved in biological processes related to different pathologies, including cardiovascular disease, Alzheimer’s disease, tumor formation, oxidative stress, and neurotransmission. To assess which biological functions were impacted by the PS, we evaluated the possible enrichment of functional gene categories (GO and KEGG terms) among the dysregulated genes. Among the most strongly enriched GO biological processes in genes dysregulated on day 3 vs. the baseline were cell death, cellular responses to stress and chemical stimuli, and cell cycle regulation. On day 14 vs. baseline, the enriched GOs were cell cycle processes (and related processes) and cellular responses to stress (Fig. 2).

**Figure 3:**
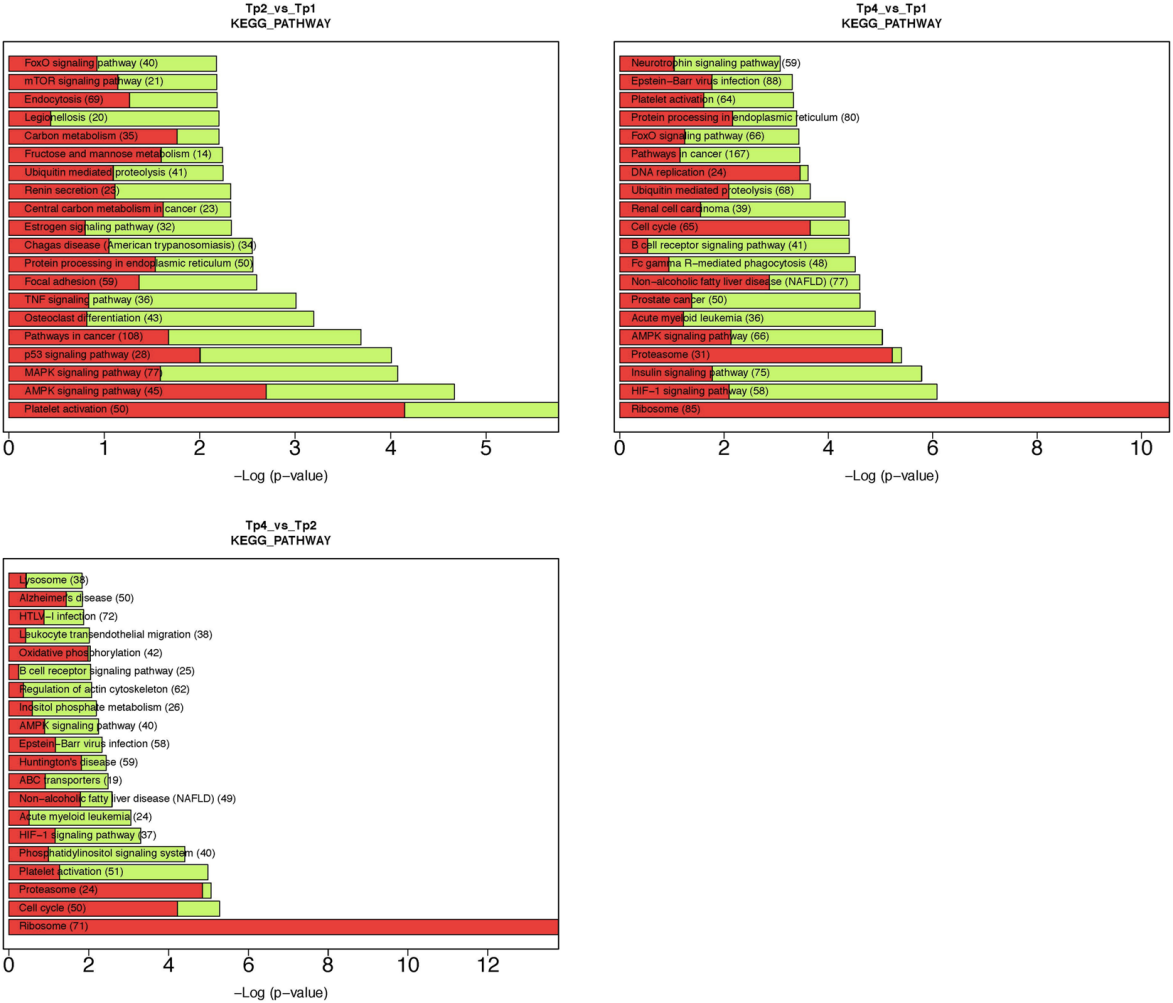
KEGG pathway enrichment analysis among genes dysregulated in response to stress based on the David tool.

Considering the Kyoto Encyclopedia of Genes and Genomes (KEGG) pathways, the dysregulated pathways on day 3 vs. the baseline were enriched for the platelet activation pathway (hsa04611), the AMPK and MAPK signaling pathways, and cancer (Fig. 3). On day 14 vs. the baseline, among the most overrepresented KEGG pathways were those related to homeostatic processes, including HIF-1 signaling (involved in oxygen homeostasis and immunological response), insulin signaling (involved in energy homeostasis), and AMPK signaling (a key regulator of cellular energy homeostasis). Other enriched pathways were related to cancer, including acute myeloid leukemia, prostate cancer, and renal carcinoma, and Fc gamma R-mediated phagocytes, which are essential for host defense because of their participation in the uptake and destruction of infectious pathogens.

### Weighted Gene Correlation Network Analysis (WGCNA) of blood transcriptomic responses to PS

**Figure 4:**
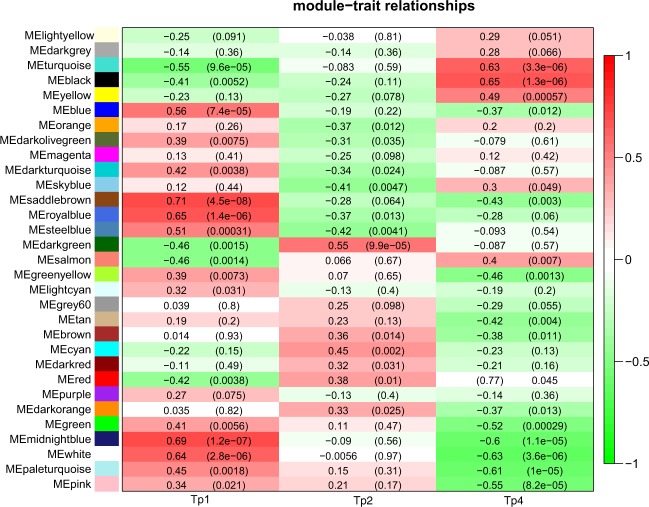
Module - time point correlation of the WGCNA modules. Each row corresponds to a module, while each column represents one time point: Tp1 i.e. baseline, Tp2 (day 3) and Tp4 (day 14). Correlation coefficients between the PC1 of a given module and specific time point are shown as color shades of each cell. The numeric value of correlation for each module-time point pair is also shown as numbers in each cell with a corresponding p-value beneath in parenthesis.

We grouped genes into modules using WGCNA (Supplementary Figure 4). We identified 31 gene modules, including 10 modules that were significantly correlated with at least one experimental time point (correl. ≥0.55, p-value < 0.001) (Fig. 4). We conducted pathway enrichment analysis for significantly correlated modules. The modules that showed the strongest correlations with a time point (correl. ≥65) were associated with rather general biological terms. For example, the saddle brown module (correl. 71 with day 0) was associated with alternative splicing. The midnight module (correl. 69 with day 0) was associated with alternative splicing and phosphoprotein. The royal blue module (correl. 65 with day 15) was associated with protein modification. The black module (correl. 65 with day 15) was associated with acetylation, cell division, mitotic processes, and mitochondrial respiratory chain functions. Finally, the turquoise module (correl.−55 with day 0 and correl. 63 with day 15) showed increased expression during the PS experiment and enrichment of phosphoprotein, acetylation, and mitotic and cell cycle processes.

**Figure 5:**
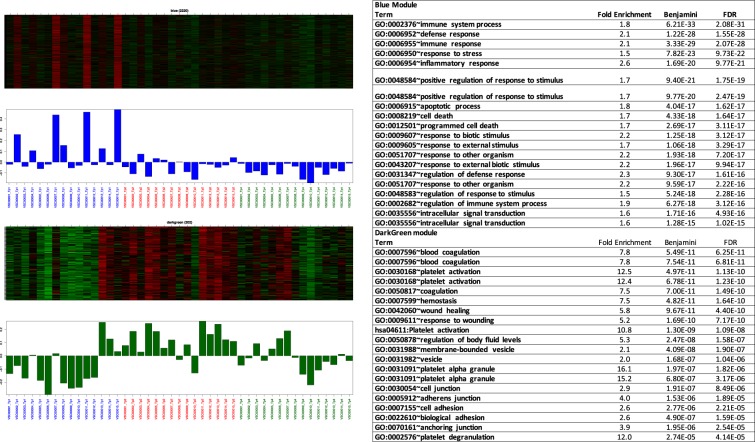
Eigengene (the 1st principal component) values of the blue and dark green WGCNA modules by increasing time points. Samples from 15 animals are organized by time points (first Tp1, then Tp 2, followed by TP4).

More specific biological terms were associated with the modules moderately and significantly correlated with the course of PS (Fig. 5). The blue module (correl. 56 with day 0) showed the enrichment of numerous terms related to immune and inflammatory functions, including the immune system process, defense response, immune response, response to stress, inflammatory response, apoptotic process, and cell death. These observations suggest that PS can impact health through the disruption of immune, inflammatory, and cell death processes. The dark green module (correl. 55 with day 3) was characterized by its transiently elevated expression on day 3; enrichment was observed for blood coagulation, platelet activation, wound healing, and response to wounding. This observation is consistent with the association of the platelet activation pathway with transient gene dysregulation on day 3 and points to increased cardiovascular risk following short-term PS.

### Stress alters cellular composition of blood

**Figure 6:**
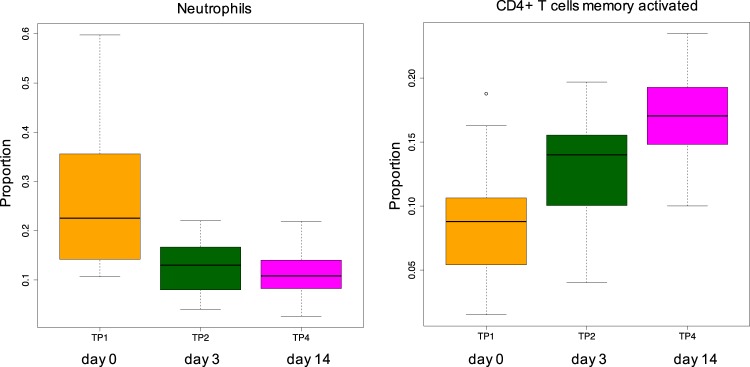
The alterations of neutrophil and activated memory CD4 + T-cell ratios across the experiment.

Blood is a heterogeneous tissue comprised of a mixture of different cell subtypes. We investigated whether stress had an effect on the composition of cell types in peripheral blood. To estimate the proportions of different cell subsets in our whole blood samples, we employed deconvolution of the bulk transcriptomic data using the Cibersort tool^49^. We compared the estimated fractions of specific cell types between day 3 and day 14 versus the baseline (day 0) (Supplementary Table 2, Supplementary Figure 5). We observed significant stress-induced dysregulation of two immune cell types, activated memory (but not resting) CD4 + T-cells and neutrophils, on day 14, the time point at which we also observed the strongest decline in the levels of the immune signaling molecules (Fig. 6). Activated memory CD4 + T-cell proportion showed a significant increase (two-tailed Wilcoxon signed-rank test p-value = 0.0008), while the neutrophil fraction showed a significant decrease (two-tailed Wilcoxon signed-rank test p-value = 0.0019) in response to stress.

## Discussion

We have proposed and validated a natural model of PS, which leverages the routine quarantine procedure of wild-born vervets, to facilitate studies of responses to displacement from a natural social and physical environment under controlled conditions. The quarantine increased the accumulation of cortisol in hair, suggesting a sustained activation of the HPA axis concordant with chronic stress and thus supporting our model of PS. Stress can induce immunoprotective (e.g., promote wound healing and eliminate infections), immunopathological, and immunoregulatory effects depending on the context and the duration of the stressor^36–40^. We observed that, among the array of conventional health biomarkers of immune signalling, glycemic health and microbial translocation, nine cytokines, chemokines, and growth factors showed a rapid decline (reaching their lowest levels on day 14), followed by a varying rate of recovery to baseline levels. Given that both classical pro-inflammatory and anti-inflammatory molecules followed this decrease-recovery pattern, we conclude that it is consistent with the transient immunosuppressive effect of stress that is characteristic of chronic stress and followed by adaptation to the novel environment.

Chronically elevated cortisol levels and stress can induce hematological responses manifesting in quantitative alterations in key cell populations in the blood, for example, due to adrenal gland disease in Cushing syndrome or due to physical and psychological stressors in humans^50,51^. In vervets, PS differently modulated the proportions of immune cells during long-term stress (on day 14). An increase was observed in the activated CD4 + memory T cell proportion which are long-lived cells^52^ that may provide a protection against previously encountered pathogens. Our results are concordant with the observation that memory CD4 + T cells were positively linked to responses to a combination of aerobic exercise and heat stress in humans^53^. A PS-associated decrease was observed in the neutrophil fraction. Neutrophils are short-lived cells and the most abundant type of granulocytes acting as the first line of defense against infection as a part of the innate immune responses. They are involved in thrombotic processes, which suppress pathogen dissemination through coagulation^54–56^. The stress-related decrease of neutrophil ratios in the vervet model of PS suggests that the PS can render the exposed individuals more vulnerable to infections. In contrast to the stress related reduction of the neutrophil ratio during the PS in vervets, the neutrophil counts were positively associated with glucocorticoid levels in various vertebrates^57^ and psychosocial stress in humans^50,58^, suggesting that they may respond distinctively per species according to type of stressors and duration of exposure. In summary, our findings evidence that a strong impact of PS on immune functions in vervets. It caused immunosuppression characterised by a decrease in a number of important immune signaling molecules, and also appeared to impair neutrophil-associated innate immune functions. On the other hand, PS may also have a beneficial effect through increasing the activation of CD4 + memory T cells, which may protect against re-exposure to pathogens.

We observed a massive dysregulation of the blood transcriptome in response to PS in vervet, including genes that are involved in reactions to various types of stressors (such as chemical compounds) or relevant for stress-related diseases. Short-term exposure to stress (on day 3) impacted the regulatory pathways of cell survival or cell death, and KEGG pathways associated with the activation of genes crucial for blood coagulation and platelet activation that play a pivotal role in cardiovascular disease^59^. Psychological stress has long been known to alter cardiovascular function and platelet activity^60,61^ and our observation is consistent with the hypothesis that stress may potentiate coronary disease pathogenesis in part via the activation of blood platelets^62^. Short-term stress was also associated with the AMPK signaling pathway, which is involved in sensing cellular energy status, and MAPK pathway playing a key role in stress-induced depression^63,64^. The genes dysregulated during long-term stress (on day 14) are involved in the pathways of various homeostatic processes, including cell cycle and cancer regulation and insulin signaling. Multiple health-related conditions are known to be associated with genes, which showed strong dysregulation (log2FC > 3) during stress exposure in vervet suggesting the relevance of the vervet model of PS to mechanisms linking human conditions with stress. For example, genes dysregulated upon short-term stress (on day 3) were linked to cardiovascular diseases *(ACTA2*), tumor processes (*SIM2*, *POU6F2*, *ADAMTS12*), inflammation and responses to microbes (*PALM3, FLG2)*, reaction to oxidative stress (*THSD4*), and Alzheimer’s disease (*COBL*); and genes dysregulated after long-term stress exposure (on day 14) included cardiomyopathies (*TNNI3*), oxidative stress (*CTSE*) and antiviral properties (*IFIT1b*), stress responses (*GRIA1*), and in neurobehavioral disorders (*SLC6A2*).

We demonstrated that the proposed paradigm of PS that models displacement and social isolation stressor in veret evoked significant stress manifested as increased cortisol production, mostly transient suppression of immune functions, and massive dysregulation of the blood transcriptome, including genes associated with a range of pathologies and phenotypes of cardiovascular and metabolic diseases, tumorigenesis, neurotransmission, and antimicrobial responses. However, we realize that stress-related responses, in particular the wide-spread effects on immune system functions (including, inflammatory biomarkers, blood cell composition, transcripts of genes involved in immune responses) we observed in vervets may be moderated by several variables that are unaccounted for here, including initial social rank and other behavioral attributes of the study animals. For example, personality traits were associated with transcriptional activity of pro-inflammatory genes in humans^65^ and social rank altered immune responses to infections in rhesus macaques^66^. Collecting baseline behavioral data in the natural habitat before the relocation to the captive setting could be considered in potential future studies. We envision that over time the vervet model of PS combined the longitudinal phenotyping based on minimally invasive procedures can facilitate well-powered investigations of the molecular, genomic and epigenetic mechanisms underlying shared and individual variable responses to stress.

## Materials and Methods

### Ethics statement

All animals in this study were wild-born vervet monkeys from a local population on St. Kitts Island that were transferred to and housed in the local NHP facility of St. Kitts Biomedical Research Foundation (SKBRF) accredited by the Association for the Assessment and Accreditation of Laboratory Animal Care under accreditation number A4384–01. All procedures used in this study (including animal capture, transfer, quarantine, and sampling) were reviewed and approved by the Axion Research Foundation IACUC under protocol number AC14118 and the UCLA Office of Animal Research Oversight ARC under protocol number 2014–075–02 before implementation. All animal procedures and sample collections were in compliance with the US National Research Council’s Guide for Care and Use of Laboratory Animals^67^ and meet or exceed all standards of the Public Health Service’s Policy on the Humane Care and Use of Laboratory Animals^68^.

### Study individuals

We acquired 15 juvenile male vervet monkeys from the wild vervet population on St. Kitts Island using a standardized, humane trapping procedure routinely used by the local primate facility (SKBRF) for animal acquisition^28^. The animals were transported to the SKBRF where they were subjected to conditions closely imitating a 28-day routine quarantine which, according to our hypothesis, represented significant PS. To longitudinally assess responses to PS, we collected biological samples and observational data over a 28-day period at six experimental time points (Tp) corresponding with a specific day (d) (±1 day) following capture. First, on day 0 (Tp1), baseline samples were immediately collected after trapping in the field before animals were transported to the SKBRF. Then, on day 3 (Tp2), day 7 (Tp3), day 14 (Tp4), Tp5 (day 21), and Tp6 (day 28), the animals were sampled under quarantine conditions (Supplementary Table 3).

On day 0, animals were trapped and rapidly immobilized (within 2–3 minutes) with a sedative (intramuscularly administered ketamine [10 mg/kg] mixed with xylazine [2 mg/kg]) and sampled within 15 minutes of capture, allowing us to significantly limit the impact of capture and sedation on gene expression and biochemical markers. The first sampling conducted in the field represented baseline conditions (day 0, Tp1). To minimize relatedness among the study subjects, only one individual per troop was acquired for this study (Supplementary Figure 6). Given the moderate sample size, we maximized the power by forming a homogeneous cohort with respect to developmental stage and sex, including only juvenile males. Co-trapped animals (not selected for our study) were intramuscularly administered the reversal agent atipamezole (0.1 mg/kg) to reverse the effects of the anesthetic and, after full recovery, were released back into the wild near the capture site so they could rejoin their troops.

Although, social status is a significant predictor of health and disease outcomes and regulator of stress perception and responses, including regulation of immune functions and their reactions to infection^23,66,69–71^, we were not able to assess pre-capture social rank in the troops in the wild, even though this variable could impact their accumulated life stress burden.

The animals selected for the PS study (15 juvenile males) were transported to the SKBRF immediately after the first sampling while they were still sedated. At arrival, the animals were placed in individual housing. Following the transfer, the animals were then sampled at four time points (day 3, 7, 14, 21, and 28, corresponding with Tp2–Tp6). To perform sampling, the animals were briefly sedated with intramuscular dexmedetomidine (0.05 mg/kg) mixed with midazolam (0.3 mg/kg). The sedative was reversed following the sampling with intramuscular atipamezole (0.1 mg/kg) mixed with flumazenil (0.5 mg/kg). The terminal sample was conducted on day 28 of the quarantine with the exception of animal VSC00015, which was sampled one day later.

### Sample collection

We collected a wide array of biological samples. PaxGene RNA blood tube samples were collected on days 0 (Tp1), 7 (Tp3), and 14 (Tp4) as a source of total RNA for transcriptomic analysis; K2EDTA blood tube samples were collected on days 0 (Tp1), 7 (Tp3), 14 (Tp5), and 28 (Tp6) to measure inflammatory biomarkers; and hair samples were obtained on days 0 (Tp1) and 28 (Tp6) to perform long-term cortisol measurements. We limited survival blood collection to 1.25% (1.25 ml/100 g) of each animal’s current body weight per week according to the IACUC recommendations for preventing anemia and dehydration. To ensure that the blood collection limits were not exceeded, we collected only whole blood for RNAseq analysis and did not collect blood for plasma on day 3 (Tp2) and thus did not perform circulating biomarker measurements at this time point.

### Cortisol levels measurements

Vervet hair is shed and regrown at a regular rate. On days 0 (Tp1) and 28 (Tp6), we collected hair samples, most of which were about 3 cm. We assessed cortisol levels using methods previously validated for vervets^31,72,73^. Cortisol concentrations were assessed in full length hair, accounting for the weight of the sample (ranging from 15 to 42 mg of hair). We did not include hair length in calculations since the results were normalized for weight. Assuming a similar hair growth rate as humans, the measured cortisol levels would be reflective of average cortisol secretion during the preceding three months.

### Circulating inflammatory and glucose metabolism biomarkers

At five time points (days 0, 7, 14, 21, 28 [Tp1, Tp3–Tp6]), we measured an array of health-related biomarkers in blood plasma using biochemical measures previously validated for wild vervets^74,75^, including sCD14 (a soluble biomarker of microbial translocation), lipopolysaccharide (LPS), and a29-plex monkey panel of immune signaling molecules, including 15 cytokines (IL-1β, IL-1RA, IL-2, IL-4, IL-5, IL-6, IL-10, IL-12, IL-15, IL-17, G-CSF, GM-CSF, IFN-γ, IP-10, TNFα), 10 chemokines (CCL-11 [eotaxin], IL-8, CCL-2 [MCP-1], CCL-22 [MDC], MIF, CXCL-9 [MIG], CCL-3 [MIP-1α], CCL-4 [MIP-1β], I-TAC, CCL-5 [RANTES]), and 4 growth factors (EGF, FGF-basic, HGF, and VEGF).

We also assessed circulating biomarkers of glucose metabolism, A1C hemoglobin, and total glycated hemoglobin GHb in whole blood at baseline day 0 (Tp1), day 14 (Tp4), and day 28 (Tp6); these measures reflect average blood glucose levels for the previous three months. For total glycated hemoglobin (GHb) testing with calculated A1c levels, we used the Primate GHb/A1c Testing kit based on high performance liquid chromatography (HPLC - boronate affinity) method (DTIL Laboratories, Inc.). Health-related biomarkers with five or more values <0 were discarded. Variables with fewer than five values <0 had negative values set to zero. A total of 16 health-related variables were analyzed (Table 1).

### Transcriptome analysis using RNA-seq

Total blood RNA was extracted from PaxGene Blood RNA tubes (Preanalytix) using PAXgene Blood RNA Kit (Qiagen). Immediately after extraction, the RNA concentrations were measured with Nanodrop, and the integrity was assessed using a bioanalyzer (Supplementary Table 4). RNA integrity number (RIN) ranged from 7.5 to 9.1, with an average value of 8.5, and concentrations ranged from 11 ng/ul to 415 ng/ul, with an average value of 127 ng/ul.

We conducted RNA-seq in blood RNA samples on day 0 (Tp1), day 3 (Tp 2), and day 14 (Tp4). For cDNA library creation, we used the TruSeq Stranded RNA protocol and 100 ng of RNA under RiboZero and Globin treatment (Illumina). We generated 120 bp-long paired-end sequencing reads using high-output flow cell lines in the HiSeq. 4000 sequencer (Illumina). Our dataset was deposited in the NCBI’s Gene Expression Omnibus (GEO) under accession number GSE120900.

### Bioinformatic analysis

RNA-seq reads were aligned to the *Chlorocebus sabaeus* reference genome (Chlorocebus_sabeus 1.1 GCF_000409795.2)^76^ using the STAR aligner^77^. Fragment counts were derived using the HTSeq program^78^. Differential gene expression analysis was conducted using the Bioconductor packages. Count data were submitted to edgeR modeling with a negative binomial distribution^79^. In both cases, we used FDR-adjusted p-values lower than 0.1. To characterize the correlation patterns among genes across the samples, we employed weighted gene correlation network analysis (WGCNA)^80^. Genes with a CPM > 0.5 in at least 3 samples were selected for constructing the signed network. We used the Database for Annotation, Visualization and Integrated Discovery (DAVID) v6.8 to understand the biological functions of the genes that were differentially expressed or co-regulated in response to PS. We used available vervet gene annotations; however, they are not as rich and detailed as annotations for the human, so in the analysis we additionally used human annotations (including KEGG pathways) available in the David tool^81^.

To estimate the relative proportions of different cell types in the whole blood samples based on the bulk blood RNA-seq profiles, we applied CIBERSORT (for quantification of 22 immune cell types)^49^, which is an established computational method of deconvolution of transcriptomic data for inferring the cell composition of complex tissues.

## Supplementary information

Supplementary Information.

Supplementary Table 1.

## Data Availability

Our RNAseq dataset was deposited in the NCBI’s Gene Expression Omnibus (GEO) under accession number GSE120900.
