# Supplementary material for: Immunosuppressive effect and global dysregulation of blood transcriptome in response to psychosocial stress in vervet monkeys (Chlorocebus sabaeus)
Source: Sci Rep. 2020 Feb 26;10:3459. doi: 10.1038/s41598-020-59934-z (PMC7044305; doi:10.1038/s41598-020-59934-z)
Supplement: Supplementary file 1 — Supplementary Information. [file 41598_2020_59934_MOESM1_ESM.pdf]

Immunosuppressive effect and global dysregulation of blood transcriptome in response to psychosocial stress in vervet monkeys (Chlorocebus aethiops sabaeus)

Anna J Jasinska1, 2,* Ivona Pandrea3, Tianyu He4, Cassandra Benjamin5, Maurice Newton5, Jen Chieh Lee1, Nelson B. Freimer1, Giovanni Coppola1,6, James D. Jentsch7

1 Center for Neurobehavioral Genetics, Semel Institute for Neuroscience and Human Behavior, The University of California Los Angeles, California, USA,

2 Institute of Bioorganic Chemistry, Polish Academy of Sciences, Poznan, Poland;

3 Department of Pathology, School of Medicine, University of Pittsburgh, Pittsburgh, Pennsylvania, USA

4 Department of Pathology, School of Medicine, University of Pittsburgh, Pittsburgh, Pennsylvania, USA

5 St. Kitts Biomedical Research Foundation, St. Kitts, West Indies,

6 Department of Neurology, The University of California Los Angeles, California, USA,

7 Department of Psychology, Binghamton University, Binghamton NY 13902

* corresponding author

Supplementary Figure Legends

Supplementary Figure 1. Hair cortisol levels per individual.

Supplementary Figure 2. Microbial biomarker levels (sCD14 and LPS) in blood plasma.

Supplementary Figure 3. Glucose metabolism biomarker levels (A1C, GHb) in blood.

Supplementary Figure 4. Gene co-expression module dendrogram generated by WGCNA. The gene dendrogram was obtained by clustering the dissimilarity based on Topological Overlap with the corresponding module colors.

Supplementary Figure 5. Blood cell type composition based on Cibersort-based deconvolution of transcriptomic data. The proportion of cell types is presented for three time points: Tp1 (day 0), Tp2 (day 3), and Tp4 (day 14).

Supplementary Figure 6. Field lab locations. Animals from 15 different troops were captured at 10 general location on the Island of St. Kitts marked with orange (N=1), red (N=2), purple (N=3) pins. One juvenile male per troop was used for this study. Image generated using Google MyMaps "Map data @2018 Google".

Supplementary Figure 1

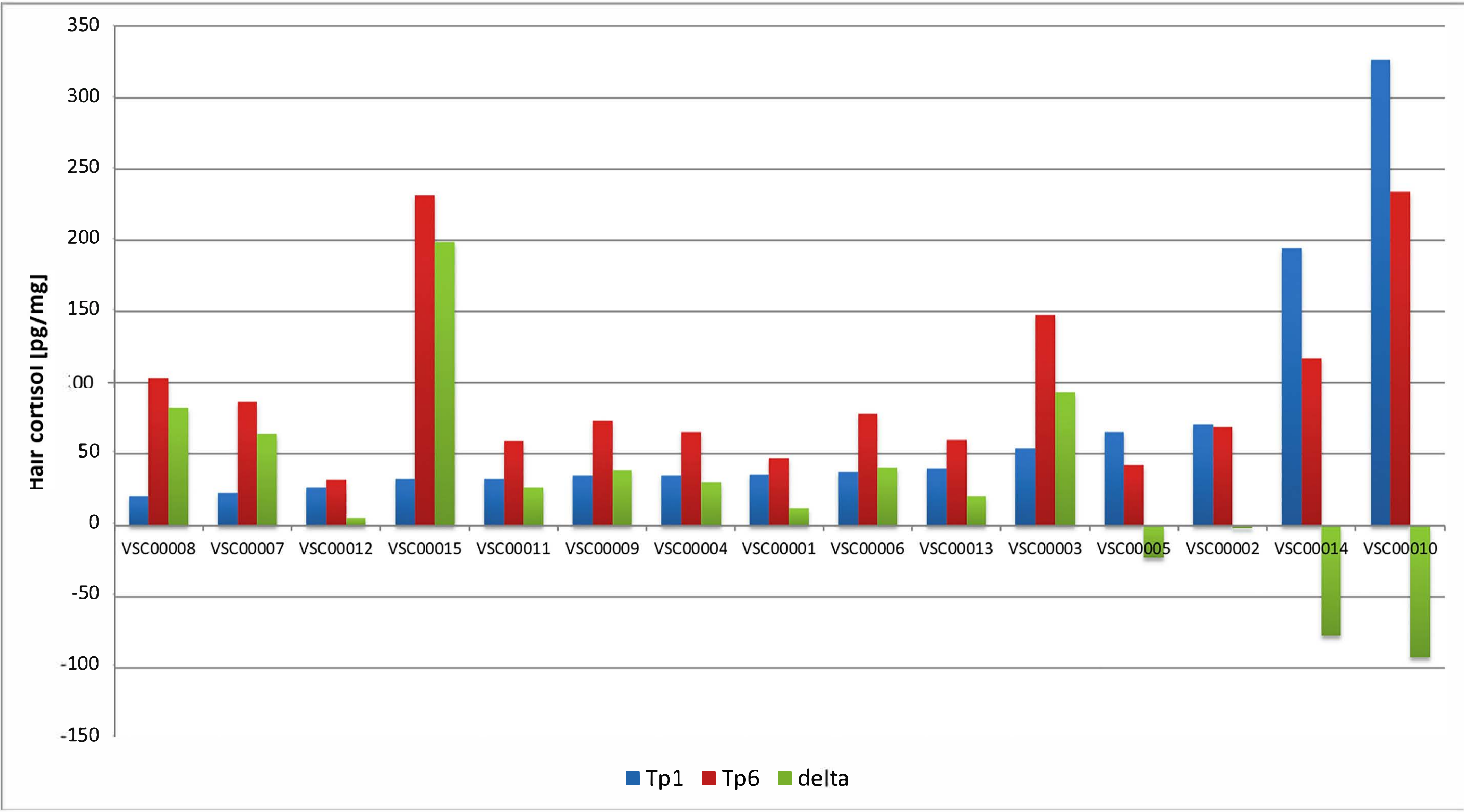

Supplementary Figure 2

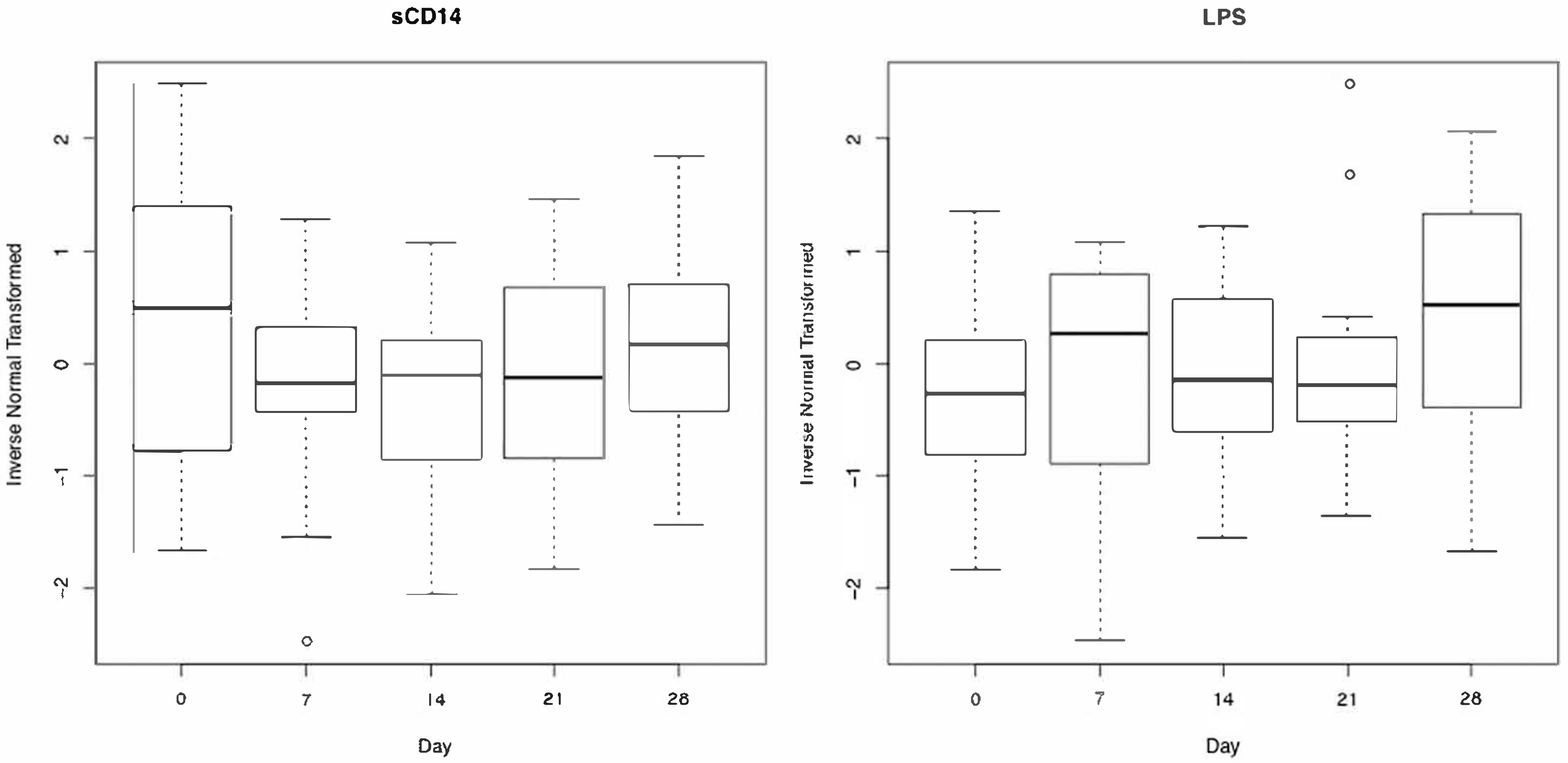

Supplementary Figure 3

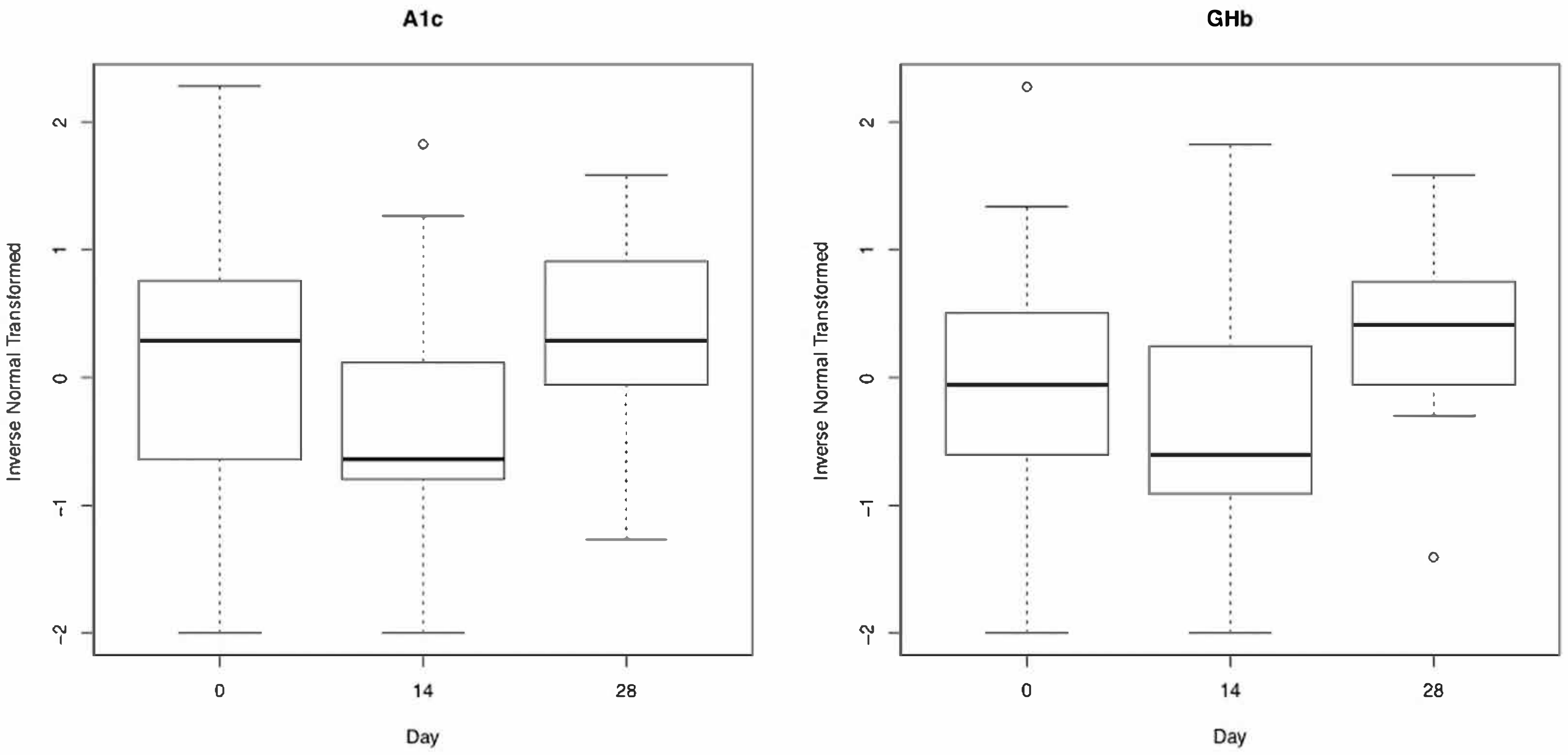

Supplementary Figure 4

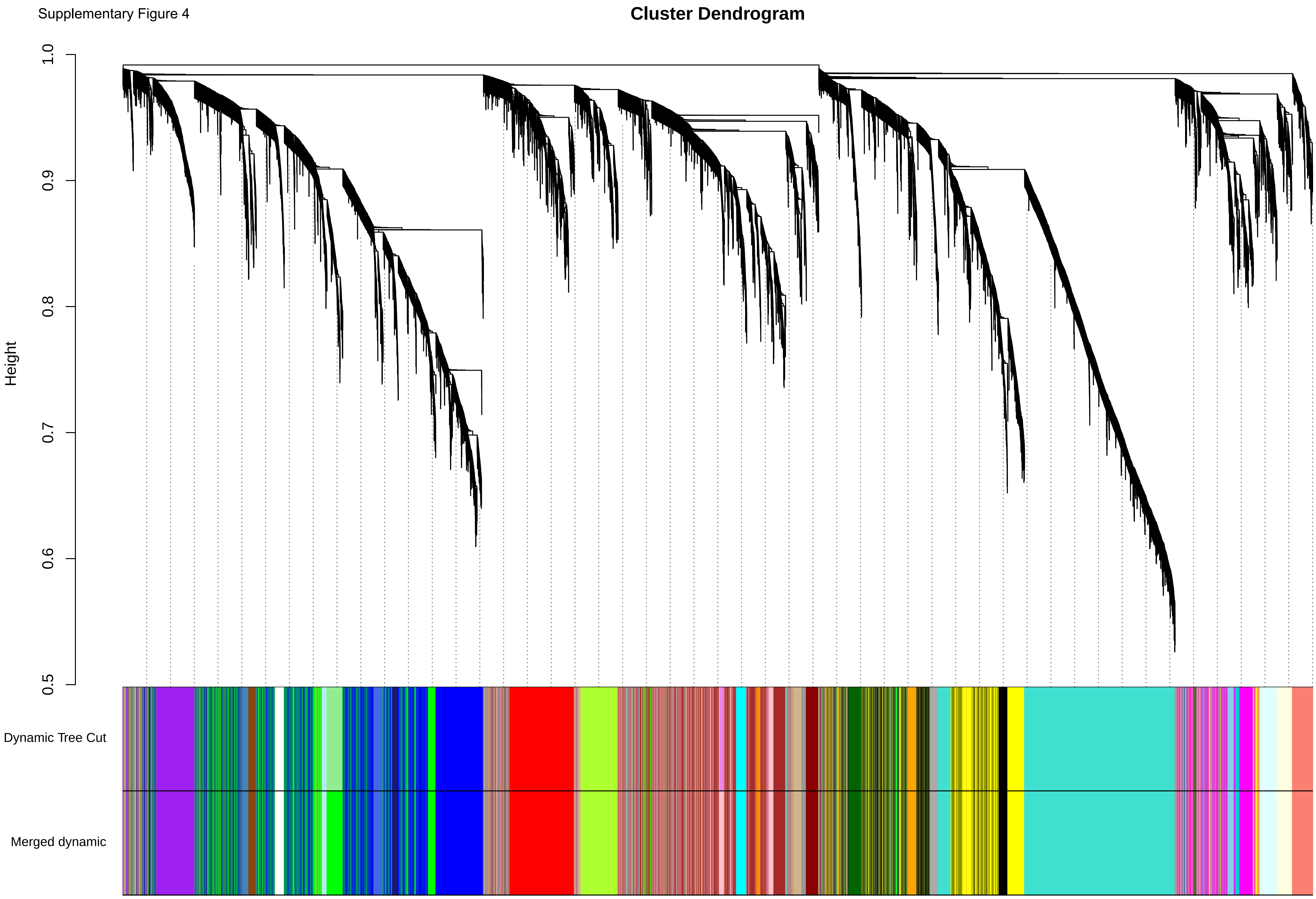

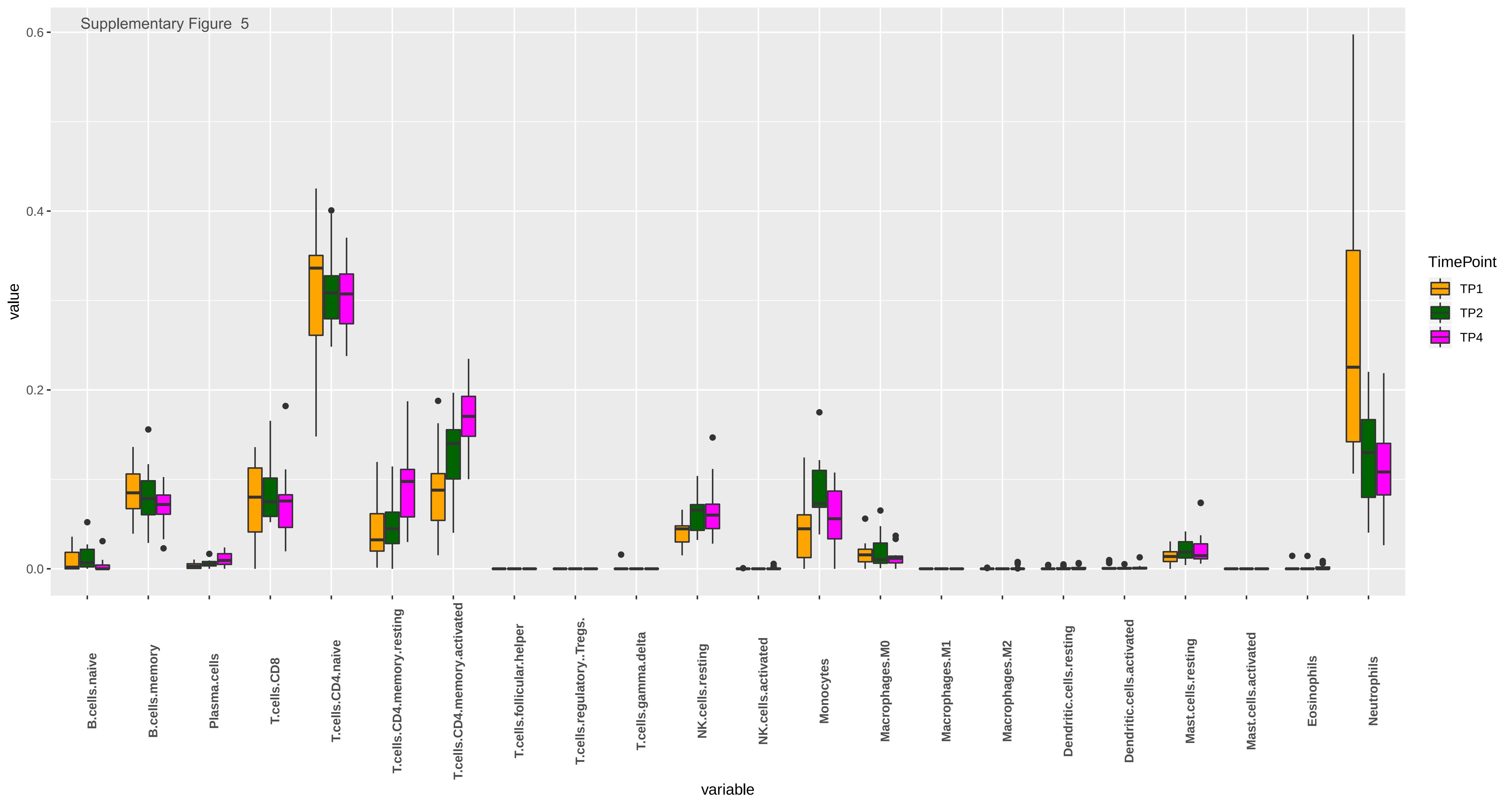
Supplementary Figure 5
value
0.6
0.4
0.2
0.0
variable
B.cells.naive
B.cells.memory
Plasma.cells
T.cells.CD8
T.cells.CD4.naive
T.cells.CD4.memory.resting
T.cells.CD4.memory.activated
T.cells.follicular.helper
T.cells.regulatory..Tregs.
T.cells.gamma.delta
NK.cells.resting
NK.cells.activated
Monocytes
Macrophages.M0
Macrophages.M1
Macrophages.M2
Dendritic.cells.resting
Dendritic.cells.activated
Mast.cells.resting
Mast.cells.activated
Eosinophils
Neutrophils
TimePoint
TP1
TP2
TP4

Supplementary Figure 6

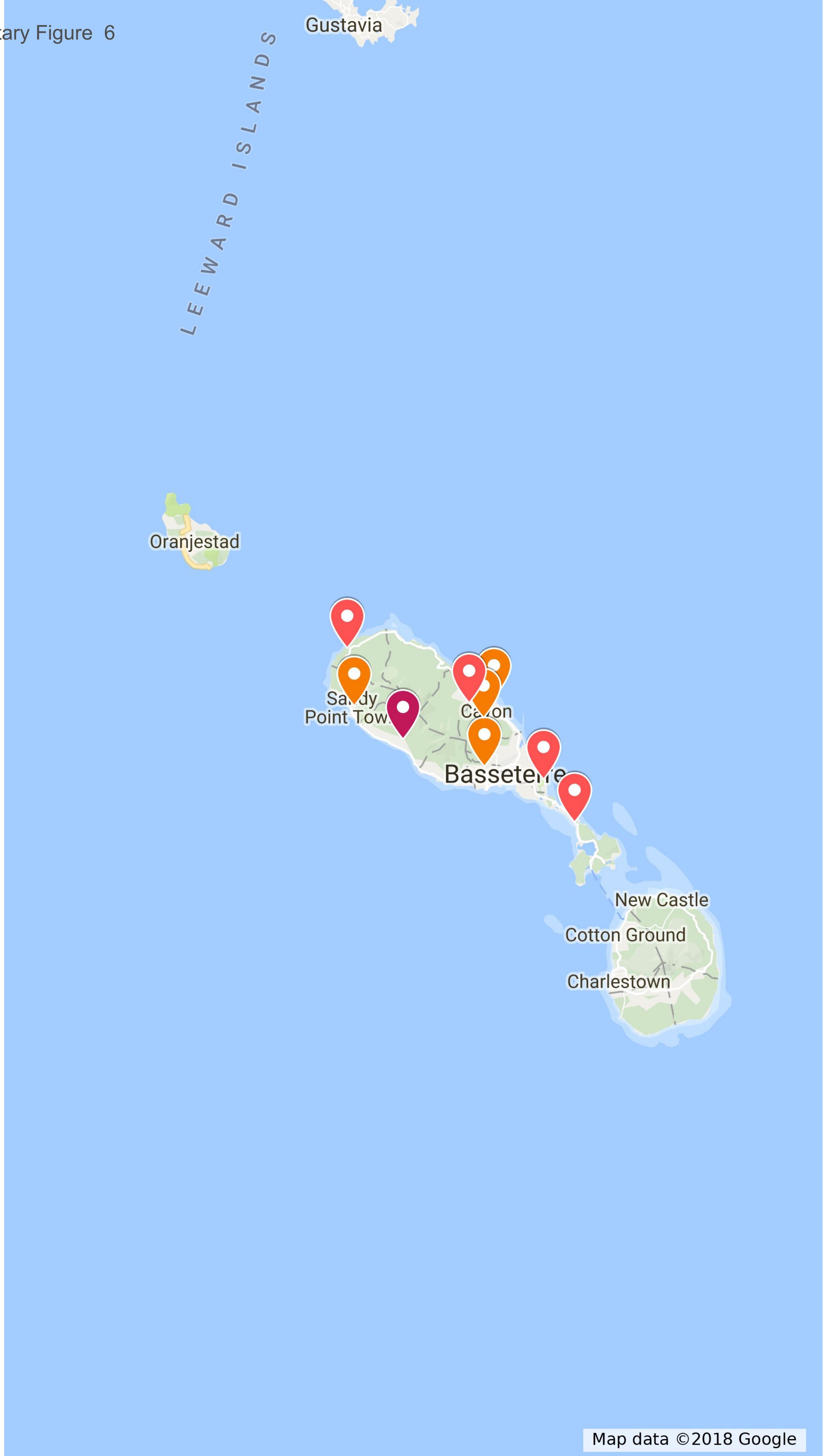

Supplementary Tables

Supplementary Table 1. DE FDR 0.05

In a separate Excel file

Supplementary Table 2. Alterations of blood cell proportions in response to PS (based on deconvolution of bulk RNAseq data using Cibersort)

Cell type and time points	Z Wilcoxon signed-rank test	two tailed Wilcoxon signed-rank test
B cells memory		
Tp2 vs Tp1	0.38	0.7039
Tp4 vs Tp1	1.75	0.0801
T cells CD8		
Tp2 vs Tp1	-0.89	0.3735
Tp4 vs Tp1	0	1
T cells CD4 naive		
Tp2 vs Tp1	-0.04	0.9681
Tp4 vs Tp1	0.33	0.7414
T cells CD4 memory resting		
Tp2 vs Tp1	-0.21	0.8337
Tp4 vs Tp1	-2.26	0.0238
T cells CD4 memory activated		
Tp2 vs Tp1	-2.31	0.0209
Tp4 vs Tp1	-3.34	**0.0008**
NK cells resting		
Tp2 vs Tp1	-2.54	0.0111
Tp4 vs Tp1	-2.83	0.0047
Monocytes		
Tp2 vs Tp1	-2.77	0.0056
Tp4 vs Tp1	-1.96	0.05
Macrophages M0		
Tp2 vs Tp1	-0.44	0.6599
Tp4 vs Tp1	1.12	0.2627

Mast cells resting		
Tp2 vs Tp1	-1.8	0.0719
Tp4 vs Tp1	-1.75	0.0801
Neutrophils		
Tp2 vs Tp1	2.6	0.0093
Tp4 vs Tp1	3.11	**0.0019**

in **bold** face p-values < 0.003

Supplementary Table 3. Longitudinal phenotypic assessments

Sample type	Assessment	Tp1 day 0	Tp2 day 3	Tp3 day 7	Tp4 day 14	Tp5 day 21	Tp6 day28
PaxGene RNA blood tube	RNA-seq	x	x		x		
Blood plasma from K2EDTA blood tube*	cytokine/chemokine panel/growth factor panel, sCD14, LPS	x		x	x	x	x
Blood plasma from K2EDTA blood tube*	A1C, GHb	x			x		x
Hair	long-term cortisol levels	x					x

* subaliquot from the same tube

Supplementary Table 4. Total RNA samples from whole blood preserved in PaxGene RNA blood tubes.

Animal ID	Time Point	Sample ID	RIN	Concentration ng/ul
VSC00001	Tp1	AAV07022	8.3	56
VSC00001	Tp2	SKN05890	8.5	265
VSC00001	Tp4	SKN06325	9	142
VSC00002	Tp1	AAV07041	8.6	42
VSC00002	Tp2	SKN05903	8.9	40
VSC00002	Tp4	SKN06341	8.9	66
VSC00003	Tp1	AAV07060	8.6	82
VSC00003	Tp2	SKN05916	8.4	133
VSC00003	Tp4	SKN06357	8.6	231
VSC00004	Tp1	AAV07079	8	33
VSC00004	Tp2	SKN05929	8.5	123
VSC00004	Tp4	SKN06373	8.3	114
VSC00005	Tp1	AAV07098	8.5	128
VSC00005	Tp2	SKN05942	8.1	85
VSC00005	Tp4	SKN06389	8.5	193
VSC00006	Tp1	AAV07117	8	127
VSC00006	Tp2	SKN05955	8.5	363
VSC00006	Tp4	SKN06405	9.1	104
VSC00007	Tp1	AAV07136	7.8	134

VSC00007	Tp2	SKN05968	8.6	272
VSC00007	Tp4	SKN06421	8.4	272
VSC00008	Tp1	AAV07155	8.3	38
VSC00008	Tp2	SKN05981	7.5	15
VSC00008	Tp4	SKN06437	8.5	223
VSC00009	Tp1	AAV07174	8.1	126
VSC00009	Tp2	SKN05994	8.1	39
VSC00009	Tp4	SKN06453	9	193
VSC00010	Tp1	AAV07193	8.5	70
VSC00010	Tp2	SKN06007	8.6	107
VSC00010	Tp4	SKN06469	8.8	125
VSC00011	Tp1	AAV07212	8.2	33
VSC00011	Tp2	SKN06020	7.7	127
VSC00011	Tp4	SKN06485	8.8	156
VSC00012	Tp1	AAV07231	7.6	29
VSC00012	Tp2	SKN06033	9.1	110
VSC00012	Tp4	SKN06501	8.8	207
VSC00013	Tp1	AAV07250	8.7	142
VSC00013	Tp2	SKN06046	9.1	54
VSC00013	Tp4	SKN06517	9.1	47
VSC00014	Tp1	AAV07269	8.2	11
VSC00014	Tp2	SKN06059	9	64
VSC00014	Tp4	SKN06533	9	241

VSC00015	Tp1	AAV07288	8.1	45
VSC00015	Tp2	SKN06072	8.8	86
VSC00015	Tp4	SKN06549	8.8	415
